# Common genes associated with antidepressant response in mouse and man identify key role of glucocorticoid receptor sensitivity

**DOI:** 10.1371/journal.pbio.2002690

**Published:** 2017-12-28

**Authors:** Tania Carrillo-Roa, Christiana Labermaier, Peter Weber, David P. Herzog, Caleb Lareau, Sara Santarelli, Klaus V. Wagner, Monika Rex-Haffner, Daniela Harbich, Sebastian H. Scharf, Charles B. Nemeroff, Boadie W. Dunlop, W. Edward Craighead, Helen S. Mayberg, Mathias V. Schmidt, Manfred Uhr, Florian Holsboer, Inge Sillaber, Elisabeth B. Binder, Marianne B. Müller

**Affiliations:** 1 Department of Translational Research in Psychiatry, Max Planck Institute of Psychiatry, Munich, Germany; 2 Max Planck Institute of Psychiatry, Munich, Germany; 3 Department of Psychiatry and Psychotherapy & German Resilience Center (DRZ), Johannes Gutenberg University Medical Center, Mainz, Germany; 4 Department of Biostatistics, Harvard University, Boston, Massachusetts, United States of America; 5 Department of Stress Neurobiology and Neurogenetics, Max Planck Institute of Psychiatry, Munich, Germany; 6 Department of Psychiatry and Behavioral Sciences, Leonard M. Miller School of Medicine, University of Miami, Miami, Florida, United States of America; 7 Department of Psychiatry and Behavioral Sciences, Emory University School of Medicine, Atlanta, Georgia, United States of America; 8 Department of Psychology, Emory University, Atlanta, Georgia, United States of America; 9 Department of Neurology, Emory University School of Medicine, Atlanta, Georgia, United States of America; 10 Phenoquest AG, Martinsried/Munich, Germany; Icahn School of Medicine at Mount Sinai, United States of America

## Abstract

Response to antidepressant treatment in major depressive disorder (MDD) cannot be predicted currently, leading to uncertainty in medication selection, increasing costs, and prolonged suffering for many patients. Despite tremendous efforts in identifying response-associated genes in large genome-wide association studies, the results have been fairly modest, underlining the need to establish conceptually novel strategies. For the identification of transcriptome signatures that can distinguish between treatment responders and nonresponders, we herein submit a novel animal experimental approach focusing on extreme phenotypes. We utilized the large variance in response to antidepressant treatment occurring in DBA/2J mice, enabling sample stratification into subpopulations of good and poor treatment responders to delineate response-associated signature transcript profiles in peripheral blood samples. As a proof of concept, we translated our murine data to the transcriptome data of a clinically relevant human cohort. A cluster of 259 differentially regulated genes was identified when peripheral transcriptome profiles of good and poor treatment responders were compared in the murine model. Differences in expression profiles from baseline to week 12 of the human orthologues selected on the basis of the murine transcript signature allowed prediction of response status with an accuracy of 76% in the patient population. Finally, we show that glucocorticoid receptor (GR)-regulated genes are significantly enriched in this cluster of antidepressant-response genes. Our findings point to the involvement of GR sensitivity as a potential key mechanism shaping response to antidepressant treatment and support the hypothesis that antidepressants could stimulate resilience-promoting molecular mechanisms. Our data highlight the suitability of an appropriate animal experimental approach for the discovery of treatment response-associated pathways across species.

## Author summary

Major depression is the second leading cause of disability worldwide. However, only one-third of patients with depression benefit from the first antidepressant compound they are prescribed. It is a fundamental problem that the outcomes of individual antidepressant treatments are still highly unpredictable. In clinical studies, discovery of biomarkers for antidepressant response is hampered by confounding factors such as the heterogeneity of the disease phenotype and additional environmental factors, e.g., previous life events and different schedules of psychopharmacological treatment, which reduce the power to detect true response biomarkers. To overcome some of these limitations, we have established a conceptually novel approach that allows the selection of extreme phenotypes in an antidepressant-responsive mouse strain. In the first step, we identify signatures in the transcriptome of peripheral blood associated with responses following stratification into good and poor treatment responders. As proof of concept, we translate the murine data to a population of depressed patients. We show that differences in expression profiles from baseline to week 12 of the human orthologues predict response status in patients. We finally provide evidence that sensitivity of the glucocorticoid receptor could be a potential key mechanism shaping response to antidepressant treatment.

## Introduction

A “one size fits all” approach is not effective or efficient in the treatment of major depressive disorder (MDD). Although it would be ideal to tailor available treatments to individual patients [1], patient-level antidepressant treatment outcomes are still highly unpredictable [2]. Identification of biomarkers predictive of individual treatment response or molecular biosignatures associated with response would dramatically improve the quality of care for MDD [3]. These biomarkers could also be expected to significantly reduce both treatment and loss-of-productivity costs. The latter become increasingly important because MDD has been shown to be the second leading cause of disability worldwide [4]. Finally, biomarkers could allow patient stratification and enable the selection of pathophysiologically distinct patient subgroups to allow optimized treatment choices based on biology. Such biomarkers could also inform the development of new interventions specifically targeting disease mechanisms in these subgroups.

Conceivably, useful biomarkers for treatment response in depression could be developed through blood-based biomarkers, including genetic approaches, although psychophysiological and neuroimaging approaches are also promising [5]. However, despite considerable efforts, including large-scale hypothesis-free, genome-wide approaches during the past years [6, 7], no biological or genetic predictors of sufficient clinical utility have been identified for routine clinical use. Thus, the most effective treatment for each patient is currently identified through a trial and error process [2].

Among the potential barriers to the development of clinically useful biomarkers in depression, the following 3 have been identified as being most important. First, current symptom-based diagnoses likely group pathophysiologically distinct patients [8], leading to considerable heterogeneity among patients diagnosed with MDD [9, 10]. Second, there are a number of confounding environmental factors such as childhood maltreatment, previous life events, disease episodes, and different psychopharmacological treatment schedules that often remain unidentified and potentially reduce the power to detect true response biomarkers. Third, genetic background, age, and sex are all factors that significantly impact transcription profiles and other laboratory measurements, as well as treatment outcome [11].

In addition to the aforementioned problems, major psychiatric disorders, including MDD, are primarily viewed as brain disorders, so the question of whether peripheral measures can be informative for treatment response to centrally acting compounds such as antidepressants continues to be matter of debate [12]. During recent years, evidence has emerged that disease- and treatment-related changes may be reflected outside the central nervous system [13, 14], revealing a potential role for appropriate animal models to support biomarker discovery in MDD. To the best of our knowledge, neither an appropriate animal experimental approach nor a translational approach systematically addressing the potential of biosignatures predicting or tracking antidepressant treatment response has been published.

To overcome some of the limitations of past approaches, we here present a conceptually novel approach that allows the selection of extreme phenotypes in an antidepressant-responsive mouse strain (DBA/2J [15]) and uses these extreme groups to identify peripheral blood biomarkers associated with behavioral treatment response, which are then tested in a human patient cohort. This strategy exploits the advantages of a murine approach for the purpose of biomarker discovery, i.e., (1) to investigate a highly homogeneous group of animals in which differences in genetic background, age, and sex can be excluded, (2) to perform biomarker discovery under conditions in which interindividual confounding environmental influences, including drug plasma and brain levels, are reduced to a minimum and controlled for, and (3) to allow correlations of peripheral biomarkers with behavior but also with peripheral and central drug concentrations, and to test the overlap of blood and brain expression profiles. We hypothesize that these standardized conditions will facilitate the identification of valid peripheral biomarkers for antidepressant treatment response and allow translation to humans.

## Materials and methods

### Ethics statement

#### Animal experimental approaches

All animal experiments were approved by the committee for the Care and Use of Laboratory Animals of the Government of Upper Bavaria, Germany (AZ 55.2.-1-54-2532-127-11). All experiments were carried out in accordance with the European Communities Council Directive 86/609/EEC.

#### Human studies

PREDiCT: The study was designed and conducted in accord with the latest version of the Declaration of Helsinki. The Emory Institutional Review Board and the Grady Hospital Research Oversight Committee gave ethical approval for the study design, procedures, and recruitment strategies (Emory IRB numbers 00024975 and 00004719).

Citalopram versus CBT: Written informed consent was obtained from all participants, with the protocol conducted as approved by the Emory Institutional Review Board and registered at clinicaltrials.gov (NCT00367341).

### Development of an animal experimental approach modeling the heterogeneity in response to antidepressant treatment

#### Conceptual background

**Fig 1:**
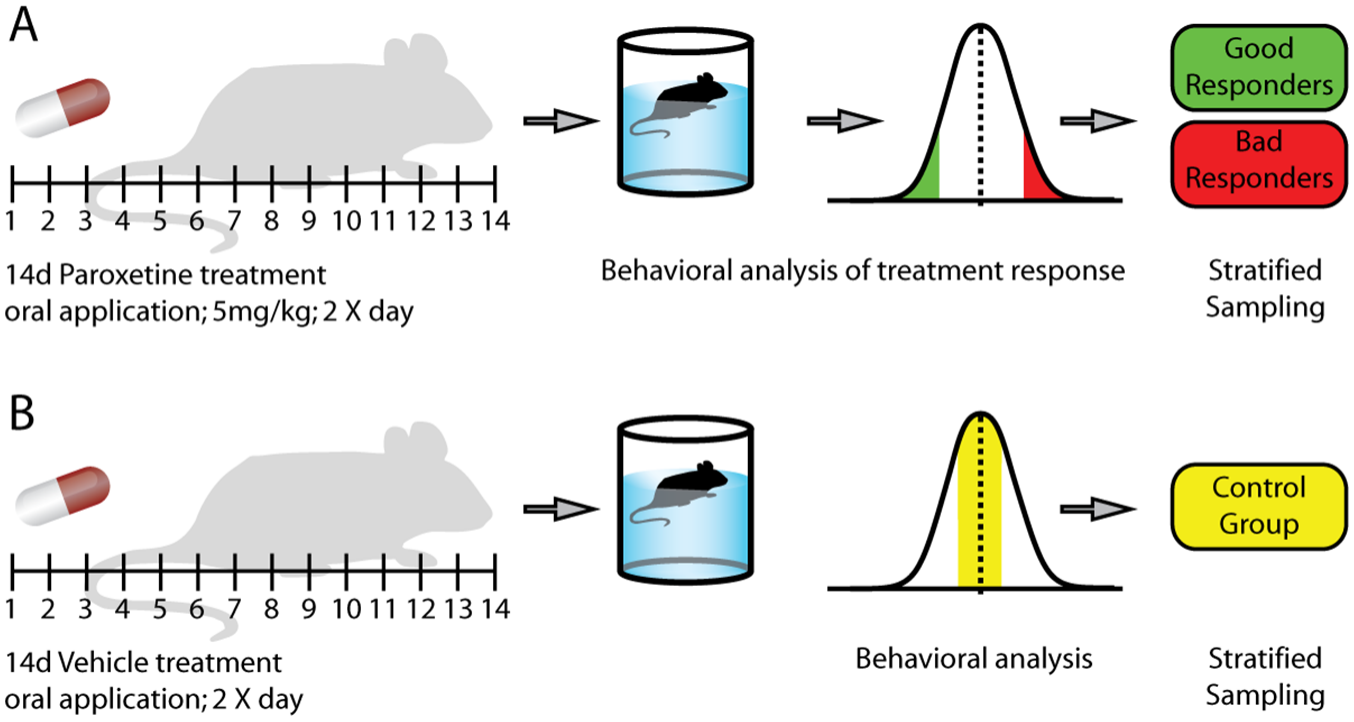
Murine approach modeling heterogeneity of treatment outcome in the FST. This figure illustrates the underlying hypothesis: in a large number of animals that are treated with an antidepressant, animals are stratified into subgroups of extremes according to their time-floating behavior. To allow for the distinction of effects truly related to the phenomenon of response (and not treatment per se), a second group of animals is treated with a vehicle under identical conditions. FST, forced swim test.

We hypothesized that within a large group of antidepressant-treated mice (antidepressant-responsive strain DBA/2J), we would be able to stratify animals into subgroups that are either responding exceptionally well (“responder”) to the antidepressant treatment or that do not show a response at all (“nonresponder,” concept visualized in Fig 1). The readout parameter for scoring antidepressant response was the forced swim test (FST, also known as Porsolt test [16]). Importantly, searching for potential predictors of early antidepressant response, a 14-day treatment was chosen according to the “early response phenotype” in clinical studies.

### Animals and housing conditions

Experiments were carried out with male DBA/2J mice (*n* = 140) from Charles River, France. On the day of arrival, the animals were 6–8 weeks old and from that day on were singly housed in standard cages under a 12L:12D cycle (lights on at 0800 h) and constant temperature (23 ± 2°C) conditions. Food and water were provided ad libitum. Pharmacological treatment of all animals started at an age of 9–11 weeks. Behavioral testing was performed at an age of 11–13 weeks. The experiments were carried out in the animal facility of the Max Planck Institute of Psychiatry in Munich, Germany, and approved by the committee for the Care and Use of Laboratory Animals of the Government of Upper Bavaria, Germany. All experiments were carried out in accordance with the European Communities Council Directive 86/609/EEC.

### Experimental design

**Fig 2:**
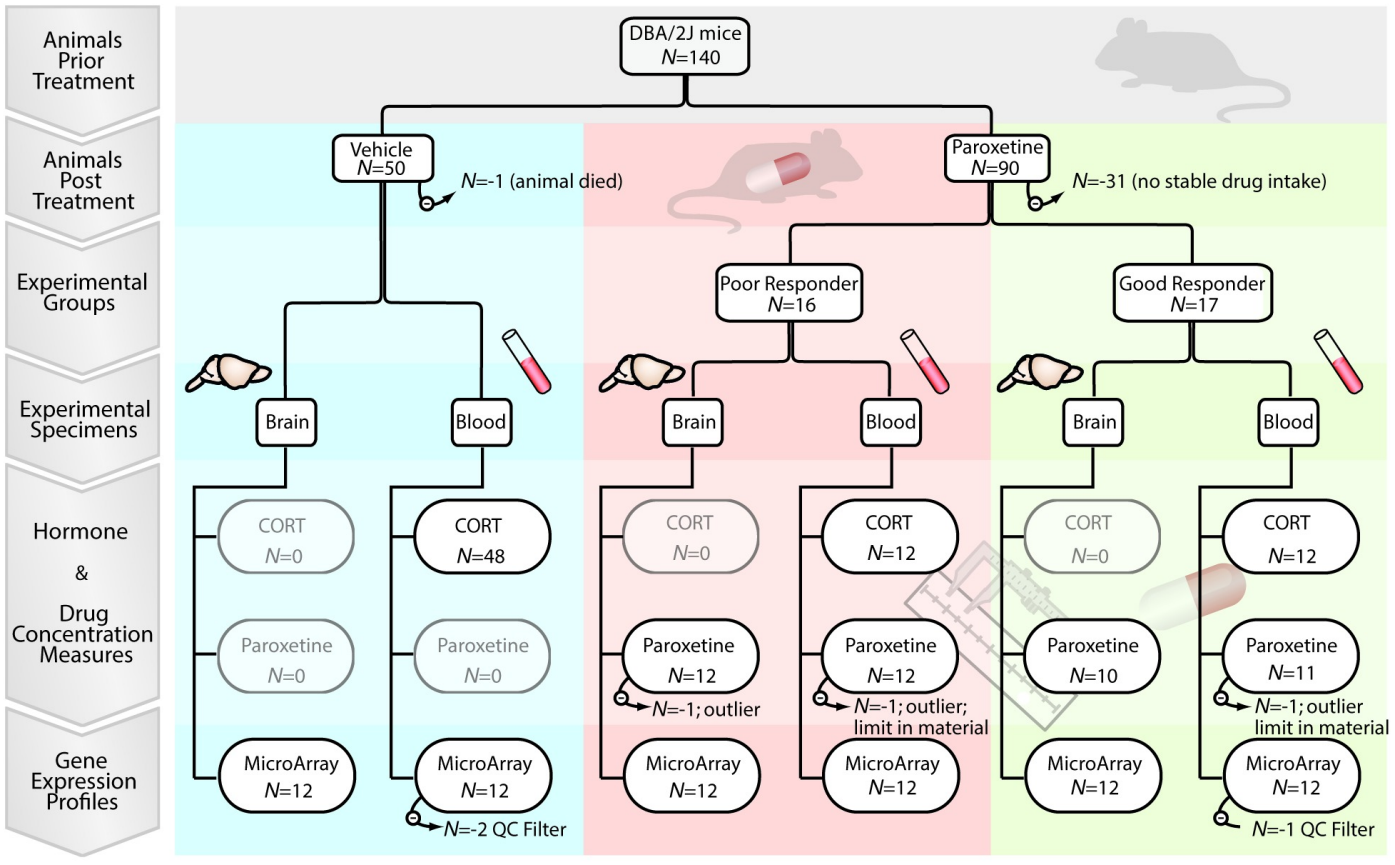
Flowchart for the entire experimental procedure. Summary figure illustrating the sequential steps of experiments and analyses applied in this study, indicating the experimental groups and group sizes for each condition. CORT, corticosterone; QC, quality control.

The sequential steps and experimental procedures are summarized in Fig 2, indicating the number of animals for each experimental group. A large number of animals were treated twice a day with either paroxetine (*n* = 90), a commonly used selective serotonin reuptake inhibitor (SSRI) antidepressant or a vehicle (*n* = 50). On treatment day 15, the animals received their last drug administration at 6 AM and were subjected to a FST 4 hours later. Directly after the FST, the animals were anesthetized with isoflurane and decapitated.

#### Oral stress-free antidepressant treatment and behavioral readout

The DBA/2J mouse strain has previously been shown to respond to oral treatment with the commonly used SSRI paroxetine under basal stress-free conditions [17], and this was the most important argument in favor of paroxetine (5 mg/kg twice daily).

Animals were randomly distributed to the vehicle or paroxetine-treated experimental group. Paroxetine (paroxetine hydrochloride; Sigma-Aldrich, Germany) or vehicle was voluntarily self-administered twice daily via customized palatable pellets (40 mg PQPellets, Phenoquest AG, Martinsried, Germany), with a concentration of 5 mg/kg body weight. To evaluate the minimum effective dosage in our mouse strain, we included an additional group of animals treated with 1 mg/kg paroxetine (paroxetine *n* = 29, vehicle *n* = 11) twice daily for comparison. Consumption was monitored on a daily basis. Animals that did not voluntarily consume the mouse pellets were excluded from the analysis.

The FST was performed on day 15 of the antidepressant treatment, 4 hours after the last drug administration, between 10 AM and noon. Each mouse was placed into a 2-L glass beaker (diameter, 13 cm; height, 24 cm) filled with tap water (22 ± 1°C) to a height of 15 cm, so that the mouse was not able to touch the bottom with its hind paws or tail. The duration of the test was 5 min. The parameters floating, swimming, and struggling were scored by an experienced observer who was uninformed regarding the treatment of the animals. Animals with the highest 20% of time floating were categorized as poor treatment responders, whereas animals with the lowest 20% of time floating were categorized as good treatment responders (Fig 1).

### Blood and brain tissue sampling procedure

Animals were anesthetized with isoflurane and killed immediately following the FST. Trunk blood was collected individually in 1.5-mL tubes. Brains were rapidly dissected and frozen at −80°C.

Due to the complex character of the study, limitations in available specimens, stringent quality control (QC), and exclusion of outlier data, we could not always achieve fully identical sample and group compositions throughout all data analysis levels. This also explains the sporadic appearance of nonconcordant group sizes, which we consider a minor but unavoidable drawback.

### Paroxetine brain and plasma concentrations

Brain and plasma paroxetine concentrations were measured after extraction by high liquid chromatography and quantifications. Paroxetine plasma concentrations were considered as a covariate in the analysis of the microarray data. For details of the respective protocols, see [18].

#### High-performance liquid chromatography

High-performance liquid chromatography (HPLC) analysis was performed using a Beckman 166 variable-wavelength ultraviolet (UV) detector (Beckman Coulter, Inc., Indianapolis, IN), a Merck L-7480 fluorescence detector (Merck KGaA, Darmstadt, Germany), and a Beckman gradient pump 126 Solvent Module (Beckman Coulter, Inc., Indianapolis, IN) equipped with a Beckman autoinjector 508 autosampler (Beckman Coulter, Inc., Indianapolis, IN) [18]. A Luna 5 μ C18(2) 250 × 4.6 mm column (Phenomenex, Torrance, CA) was used for separation; column temperature was set at 60°C and the flow of the mobile phase was 1.0 mL/min. For chromatographic analysis of paroxetine and its metabolites, a mobile phase gradient was used and combined with determination of the substances and their metabolites by UV absorption or fluorescence. The coefficient of variance was less than 15% for the different methods.

#### Quantification

Calibration of plasma samples was performed using spiked samples at different concentrations [18]. Quantification was done by calculating the analyte: internal-standard peak-area ratio. In addition, a regression model was fitted to the peak-area ratio of each substance to internal standard versus concentration.

### Corticosterone plasma concentrations

For determination of brain tissue concentrations of paroxetine, tissue from the cerebellum was dissected and rapidly frozen on dry ice. The remaining trunk blood of each animal was collected in labeled 1.5-mL EDTA-coated microcentrifuge tubes (Kabe Labortechnik, Nümbrecht, Germany). All blood samples were kept on ice until centrifugation at 8,000 rpm at 4°C for 15 min. After centrifugation, the blood plasma was transferred to new, labeled 1.5-mL microcentrifuge tubes. All plasma samples were stored frozen at −20°C until the determination of corticosterone by radioimmunoassay (MP Biomedicals, Santa Ana, CA; sensitivity, 6.25 ng/mL).

### Statistics for data obtained in in vivo experiments

The data presented are shown as means + standard error of the mean, analyzed by the commercially available software SPSS 16.0. For comparing 2 independent groups, data were analyzed with 2-tailed, independent samples Student *t* test in case of normal distribution of the data; otherwise, nonparametric comparisons were applied (Mann–Whitney *U* test). For variables with more than 2 groups, 1-way ANOVA was performed followed by Bonferroni post hoc testing. Correlations were analyzed with a 2-tailed, bivariate Pearson’s correlation analysis. As nominal level of significance, *p* < 0.05 was accepted. Values outside the 95% confidence interval (CI) were defined as statistical outliers and excluded from the analyses.

### For RNA extraction

Part of the blood was processed according to the PAXgene blood miRNA Kit manufacturer’s instructions. Briefly, 350 μL of freshly collected trunk blood was immediately transferred into 1.5-mL tubes filled with 966 μL PAXgene solution (RNA stabilizer reagent), gently inverted 10 times, incubated at room temperature (RT) for 2–24 hours, and then stored at −20°C before ribonucleic acid (RNA) isolation. Volume ratio of RNA stabilizer reagent to blood samples was kept at 2.76, according to the manufacturer’s protocol.

#### Whole blood RNA globin reduction

Blood consists of a heterogeneous cell population of erythrocytes, granulocytes, and other peripheral mononuclear cells (PBMC). This heterogeneity makes it difficult to detect differences in gene expression levels. Furthermore, it is worth mentioning that blood consists of a high amount of globin mRNA transcripts. This high amount of globin mRNA transcripts can mask differences in other mRNA transcripts. After the RNA isolation from whole blood, we applied the Ambion GLOBINclear-Mouse/Rat Kit. Globin reduction was performed according to the manufacturer’s protocol. Input RNA was quantified before the globin reduction with a Nanodrop spectrophotometer. Shortly, custom biotinylated complementary oligonucleotides were mixed with globin RNA sequences from RNA isolated out of whole blood and then annealed the oligonucleotides to α- and β-globin transcripts. Streptavidin-coated paramagnetic beads were added to bind the biotinylated duplexes and therefore removed the captured globin transcripts from the preparations of total RNA. This globin-reduced RNA was then further processed and amplified and then used for the microarray experiments.

### Gene expression profiling in mice

#### Microarray analysis

After stratification of mice into good versus poor responder subgroups according to the abovementioned phenotypic criteria, gene expression profiling by means of whole-genome gene expression microarrays (MouseWG-6 v2.0 Expression BeadChip Kit, Illumina) was performed on globin-depleted RNA extracted from 3 groups of animals: good treatment responders (*n* = 12), poor treatment responders (*n* = 12), and vehicle-treated animals (*n* = 12) (see Fig 2 for an overview of the groups). For comparison of transcriptome profiles of peripheral blood and brain, prefrontal cortex (PFC) was punched from cryosections of the same animals using a binocular microscope.

### Gene expression profiling in mice

#### RNA quantification and QC

Globin-depleted total RNA was quantified with a Nanophotometer (Nanodrop 2000, Fisher Scientific, Waltham, MA) and both quantified and quality controlled by capillary gel electrophoresis (2100 Bioanalyzer, Agilent Technologies, Santa Clara, CA; RNA 6000 nano Assay, Agilent Technologies, Santa Clara, CA). The obtained RNA integrity numbers (RIN) were greater than 7.5 in all total RNA samples derived from blood before globin depletion and dropped slightly after globin depletion (RIN > 6.3 for all samples that were further analyzed).

#### RNA amplification and labeling

Globin-depleted RNA was labeled and linearly amplified to cRNA in a commercial form of the classical procedure by Eberwine. As input for the Illumina TotalPrep-96 RNA Amplification Kit (Life technologies, Carlsbad, CA), 250 ng of RNA was used, and sample processing followed the manufacturer’s protocol exactly. cRNA was again quantified and quality checked as performed with total RNA. All samples underwent photometric analysis (Epoch Spectrophotometer with Take3 Trio Micro-Volume Plate, BioTek Instruments GmbH, Bad Friedrichshall, Germany) and a selected cross section of the samples was additionally checked on the Bioanalyzer (Agilent Technologies, Santa Clara, CA).

#### Microarray hybridization

Following the manufacturer’s instructions (WGGEX Direct Hybridization Assay Guide 11322355A), 1,500 ng of labeled cRNA was hybridized onto whole-genome gene expression microarrays (MouseWG-6 v2.0 Expression BeadChip Kit, Illumina, San Diego, CA). Sample randomization and alternating processing between the experimental groups were applied in order to avoid technical bias being correlated with group comparisons.

#### Microarray scanning and QC

Microarrays were scanned and intensity extractions were computed using a BeadArray Reader (Illumina, San Diego, CA) via the BeadScan Software with activated internal outlier detection and a scan factor of 1 (PMT = 478; PMTFactor = 1). The extracted bead summary data provide gene expression levels from 45,281 array features per sample. QC of microarray data was based on visual inspection of scan images, data distributions, internal Illumina controls, pairwise scatter plots, and statistical outlier detection of samples. Thereby, 3 samples (1 from the good responder group and 2 from the control group) were excluded from further analysis.

#### Microarray data processing and analysis

For the samples fulfilling QC criteria, bead summary scan data were filtered for detected probes with p-detection < 0.05 in at least 4 samples in the whole data set. Variables remaining numbered 20,412 and were variance stabilizing normalization (vsn) transformed and normalized using the bioconductor R package “beadarray.” Normalized and filtered data were imported into Qlucore Omics Explorer 2.3 (Qlucore, Lund, Sweden) for exploration of batch effects and for inference testing. Principal component analysis was used to identify batch effects and artifacts according to correlations of the sample structure with putative confounders. Consequently, a mild technical bias was removed by using bead chip ID as a covariate in ANOVA from blood samples analyses. Paroxetine concentrations were used as a second covariate to remove unwanted variance introduced by differences in the bioavailability of paroxetine. For some animals, blood paroxetine concentrations were not available due to limitations in total blood amount. To impute those missing values, 4 blood concentrations used in the differential gene expression analysis were predicted from regressing paroxetine concentrations from brain against blood. In the brain microarray data, covariates were paroxetine concentration in brain, and “amplification batch.” For correlation analyses of paroxetine concentrations with microarray data, paroxetine concentration as covariate was omitted.

For inference testing, variables (individual microarray probes) were further filtered for variance of >5%, which left 4,966 variables for the good versus poor responder comparison and 6,664 for the paroxetine versus vehicle comparison. Statistical tests of microarray data were based on 2 group comparisons using ANOVA with Benjamini-Hochberg based false discovery rate (FDR)-analogue *q*-values (*q* < 0.1 and *q* < 0.05).

Annotation of microarray probes was done using the manufacturer’s annotation file (MouseWG-6_V2_0_R2_11278593_A.bgx).

#### Visualization of microarray results

Microarray results were visualized as a volcano plot; all 4,966 microarray probes that have been filtered for detection over background and variance in the paroxetine blood data have been plotted. The x-axis displays the difference in residual gene expression for normalized microarray data (variance stabilizing normalization, vsn). The y-axis indicates the negative value of log 10 transformed *q*-values.

Gene regulation pattern analysis between tissues was performed using the R “stats” package (functions: heatmap, dendrogramm, hclust, and dist). All significant probes from blood were mapped to the brain array results. After removing probes with missing data in one of either tissue, 214 microarray probes remained for the analysis. Clustering was only performed for microarray probes that are represented as rows in the plot. The agglomerative clustering is based on the method “complete” and the use of a distance matrix with euclidean distances as a dissimilarity parameter.

### Impact of blood cell proportions in mice gene expression profiles

We assessed whether the observed gene expression profiles of good treatment responders and poor treatment responders were related to changes in blood cell proportions in the mice using CIBERSORT [19]. The input reference matrix of expression signature profiles of mouse tissue was obtained using ImmuCC [20]. These statistical tools infer proportions of 25 types of immune blood cell types.

### Gene expression transcripts of antidepressant treatment response tested in a human sample

To assess the relevance of the gene expression transcripts for antidepressant response differences in humans, we tested their predictive ability to classify response status in a human sample. The sample (*n* = 86) consisted of a subset of MDD patients treated with antidepressant drug treatment over 12 weeks from 2 samples recruited at Emory University School of Medicine (*N* = 74 from [21] and *N* = 12 from [22]). In both studies, patients followed a similar protocol and were randomized to either antidepressant drug treatment or cognitive behavior therapy (CBT), with the difference that patients were randomized to CBT, duloxetine, or escitalopram in PReDiCT [21] and to CBT or escitalopram in [22]. Only the subset of patients in the antidepressant treatment group with sufficient RNA quality at both time points was included in this study. Please see S2 Table for a brief synopsis of demographic and clinical parameters on the patients from clinical studies. Depression severity was assessed at baseline and week 12 using the Hamilton Depression Rating Scale (17 items, HDRS-17). In both samples, blood was drawn at baseline and after 12 weeks of treatment into Tempus RNA tubes (Applied Biosystems).

RNA was isolated from peripheral blood in a 96-well format using the magnetic bead-based technology MagMAX for Stabilized Blood Tubes RNA Isolation Kit, compatible with Tempu Blood RNA Tubes (Ambion/Life Technologies, Carlsbad, CA; cat# 4451893). RNA was quantified using the Nanophotometer, and quality checks were performed on the Agilent Bioanalyzer (Agilent Technologies, Santa Clara, CA). Only samples with RIN ≥ 6 with clear 18S and 28S peaks on the Bioanalyzer were used for amplification; the average RIN was 6.3 (SD of 0.668). RNA was further processed for generation of biotin-labeled amplified RNA using the Amplification Kit (Ambion/Life Technologies, Carlsbad, CA; cat# 4393543). cRNA was hybridized to Illumina Sentrix Arrays HT-12 v4.0 arrays using the Illumina TotalPrep-96 RNA (Life technologies, Carlsbad, CA) and incubated overnight for 16 hours at 55°C. Arrays were washed, stained with Cy3 labeled streptavidin, dried, and scanned on the Illumina BeadScan confocal laser scanner (Illumina, San Diego, CA). QC was performed using the bead-array package in R for 86 samples and 47,282 probes. Probes with p-detection values of <0.01 in at least 10% of the samples in the whole data set were removed. Remaining probes were normalized and transformed using the vsn package in R. Not all samples were hybridized on the same batch, and thus we corrected for chip number using COMBAT. A total of 17,725 transcripts and 86 samples remained after QC.

For the full drug-treated sample, 63 patients were classified as responders and 23 as nonresponders, according to percent changes in HDRS-17 scores from baseline to week 12 (≥50% or <50% change, respectively).

Mouse gene expression transcripts (*n* = 259) resulting from the microarray analysis and described in S1 Table were mapped to their human orthologue genes present in the Illumina HT-12 arrays (*n* = 241). Because some genes are represented by more than one probe, 288 probes were included in final analyses. Prediction models were built as soft margin support vector machines for classification using the e1071 packages in R with the parametrization gamma = 0.001; cost = 10. Further analyses included only mouse transcripts at FDR of 5% (*n* = 85). These were also mapped to their human orthologue genes (*n* = 77); 66 genes passed QC in the human study, which were represented by 92 probes. The sample was equally divided into training and test data sets for each of the analyses (probes at *q* < 0.1 and *q* < 0.05). Gene expression repeated measures from the patients at baseline and week 12 were available; we computed the absolute difference between the expression levels of the transcripts between those time points and tested whether these differences were able to predict response to antidepressant treatment in the test data set. We permuted the response-status labels 10,000 times in the training data set and predicted the response status in our test data. In addition, we compared the obtained prediction accuracy of our selected classification features against 1,000 classification models derived from randomly sampled features. Random feature sets also consisted of absolute difference in expression between baseline and week 12 of treatment and were size matched to the selected feature set. Those data were the input for soft margin support vector machine training and testing as indicated above.

### Impact of blood cell proportions in human gene expression profiles

We assessed whether the observed gene expression changes in responders versus nonresponders were related to changes in cell proportions in the human samples using the Cell-type Computational Differential Estimation CellCODE R package [23]. Separate components for neutrophils, T cells, stimulated T cells, NK cells, dendrite cells, stimulated dendrite cells, monocytes, B cells, and plasma cells were extracted using markers from the IRIS reference data set provided by CellCODE.

### Pathway analysis

Two available tools have been used for pathway analyses: DAVID (https://david.ncifcrf.gov/) and Pathway-Express [24].

Both tools were used with a list of gene symbols previously shown to be significantly regulated (*q*-value < 0.1) with differential paroxetine response and interrogated with respect to a custom background that contained all microarray probes that have been used for computing inferential statistics. The background contained probes that passed our detection and variance filters.

### Functional overlap of differential paroxetine response with dex-regulated genes

To determine the function overlap of differential paroxetine response with dex-regulated genes, we used data from a microarray experiment in male C57BL/6N mice at an age of 12 weeks (mean body weight 26.8 ± 0.1 g), in which animals were treated with 0.1 mg/kg dexamethasone i.p. or vehicle (*N* = 10 and 10) between 0900 and 1100 and sacrificed 4 hours later [25]. Trunk blood was collected into microcentrifuge tubes containing PAXGene RNA stabilizer solution and frozen at −20°C. RNA was then extracted using the PAXgene blood miRNA kit (PreAnalytiX), amplified using the Illumina Total Prep 96-Amplification kit (Life Technology), and then hybridized on Illumina MouseRef-8 v2.0 BeadChips.

Analyses were performed using custom scripts in R. First, a common content for both microarray data sets was generated based on Illumina “Probe Ids.” Within that common content, differentially expressed microarray probes were identified for both contrasts using an FDR threshold of *q* < 0.1. For the differential paroxetine response, 179 probes passed that threshold. Then, the number of array probes overlapping with dex regulation by chance was determined using 100,000 random sampled gene sets of size *N* = 179. For each trial, the overlap to the fixed dex-regulated gene list (*N* = 1,882) was determined and all the results were finally compared to the overlap of paroxetine response genes with dex-regulated genes; this was done by counting the number of sampled sets that showed higher overlap (>134) than the differential gene list.

In addition, a 2 × 2 contingency table was computed for dex regulation and paroxetine response and these numbers were further used to perform a hypergeometric test.

Calculation of statistical significance for a possible directionality of gene regulation was performed using a binomial test.

## Results

### Modeling heterogeneity in antidepressant treatment response in mice

In order to detect the minimum effective dosage of paroxetine for the DBA/2J strain, 2 paroxetine concentrations (1 mg/kg body weight or 5 mg/kg body weight, twice daily) were tested in a pilot study. The lower paroxetine concentration (*n* = 29) failed to produce a significant behavioral treatment effect in the FST. The only parameter that was altered with the 1 mg/kg dosage was body weight (T_39_ = −2.490, *p* < 0.05). Behavioral data, neuroendocrine measurements, and body weight are shown in S1 Fig.

**Fig 3:**
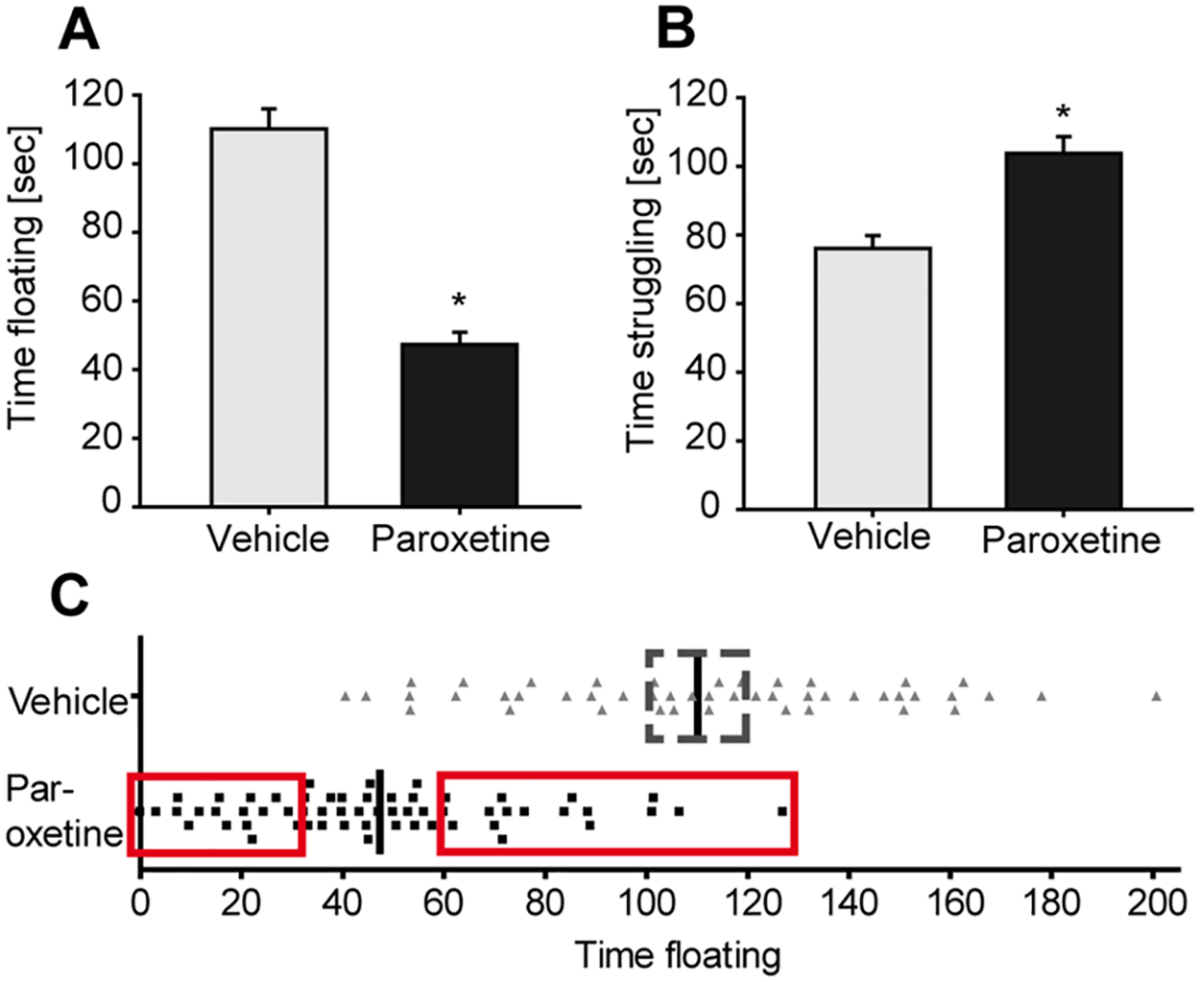
A 14-day paroxetine treatment significantly reduces depression-like behavior in the FST. (A) Paroxetine-treated animals showed a reduction in time floating compared to vehicle-treated animals. (B) Paroxetine significantly increased active coping strategies, i.e., time struggling, compared to vehicle-treated animals. (C) Identification of different responder groups according to their performance in the FST. Animals indicated in the red squares are referred as good and poor treatment responders. Animals that showed a very high time floating represent the poor treatment responder, whereas animals that showed a very low time floating represent the good treatment responder. Animals indicated in the dotted-line square represent internal control groups. Animals within the vehicle-treated group served as a vehicle-treated control group. * indicates significant difference between the vehicle-treated control group and the paroxetine-treated group, *p* < 0.000. All raw data for Fig 3 are available in S2 Data. FST, forced swim test.

A dosage of 5 mg/kg evoked a significant antidepressant-like response in the FST (Fig 3). The following data were all collected from animals treated with 5 mg/kg paroxetine, which we considered to be the minimum effective dosage for the DBA/2J strain.

#### Behavioral heterogeneity of paroxetine-treated mice allows stratification into good versus poor treatment responders

Paroxetine-treated animals showed a significant reduction in the time floating (Fig 3A) (T_80.701_ = 9.157, *p* < 0.000) and a significant increase in the time struggling (Fig 3B) (T_102.624_ = −4.496, *p* < 0.000). Furthermore, there was a large heterogeneity within the paroxetine-treated animals (Fig 3C), thus allowing selection of extreme subgroups (animals with low time floating were considered “good responders;” animals with a high time floating were considered “poor responders” [20% extremes]).

#### Physiological and neuroendocrine parameters

Paroxetine-treated animals gained significantly more body weight compared to the vehicle group (T_105_ = −8.356, *p* < 0.000) (S2 Fig). However, there was no significant difference in body weight gain between the responder and nonresponder groups (S2 Fig).

Plasma corticosterone concentrations were assessed directly after the 5-minute FST. No significant difference in corticosterone plasma concentrations was detected between vehicle- and paroxetine-treated animals (S2 Fig) or between the subgroups of good- versus poor-responding animals (S2 Fig).

### Paroxetine plasma and brain concentrations

There was no significant difference in plasma paroxetine concentrations between the good and poor treatment responder (*p* = 0.19). For paroxetine brain concentrations, a significant difference between good and poor responders could be detected (*p* < 0.05) (S3 Fig). Paroxetine brain and plasma concentrations were closely correlated (*r* = 0.94; *p* < 0.0001) (S3 Fig). Despite the lack of statistical association, we included plasma paroxetine concentrations as a covariate in further analyses on the transcriptome profiles in peripheral blood samples. Brain paroxetine concentrations were used as covariates in analyses of PFC samples.

### Microarray analysis in peripheral blood

To identify signature gene expression profiles characteristic of the animals’ responder status, gene expression data sets of vehicle-treated animals, good responders, and poor responders were created by whole-genome gene expression microarray analysis on blood samples and analyzed (*n*[vehicle] = 12, *n*[good] = 12, *n*[poor] = 12). We evaluated both treatment effect and response status with respect to antidepressant treatment and with respect to paroxetine plasma concentrations. We also investigated whether paroxetine brain or plasma concentrations might have an effect on gene expression levels. Linear and quadratic regression analyses did not reveal any microarray probe that showed significant correlations with the related plasma paroxetine levels when controlling for multiple testing. No significant influence of paroxetine concentrations on gene expression profiles was observed. Nevertheless, identified technical batch effects in the data and measured paroxetine drug concentrations in blood were used as covariates in an ANOVA-based statistical model.

**Fig 4:**
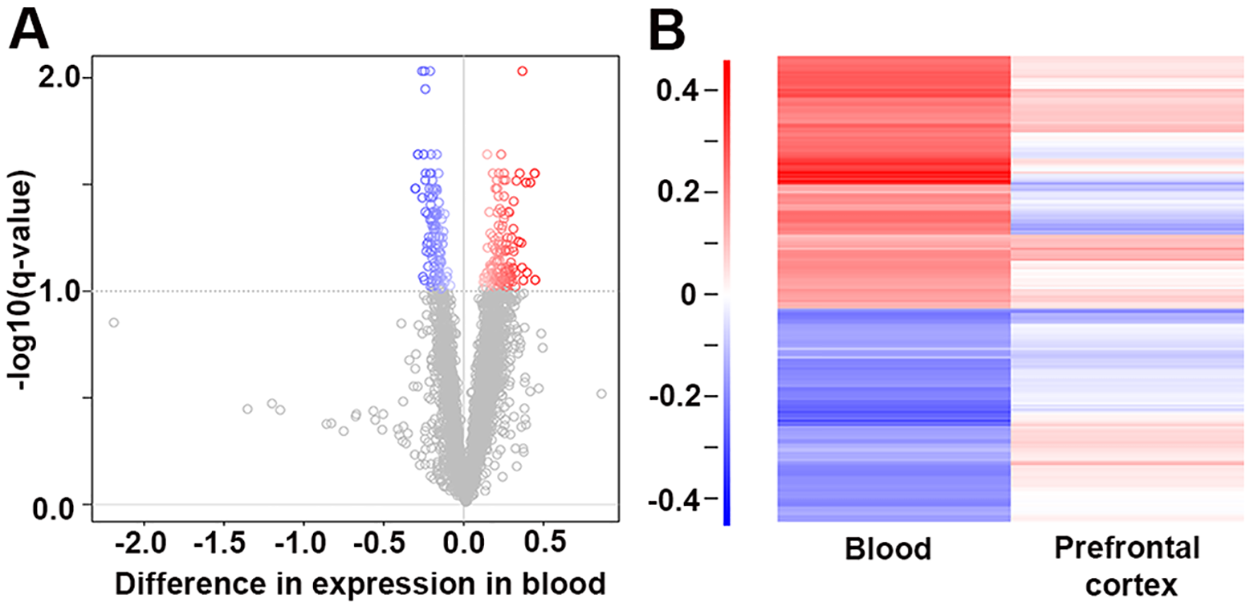
Differential gene expression in animals stratified for behavioral treatment response to chronic paroxetine treatment. (A) Volcano plot showing results from blood samples. The biological effect size (difference in expression) is plotted against statistical significance (as negative log 10 transformed values of the FDR-based *q*-value). Regarded as significantly regulated, 259 probes with *q*-values < 0.1 are colored according to their difference in expression. (B) Heat map comparing patterns of differential gene expression in blood and prefrontal cortex. Each row in the plot represents the difference in expression of 1 microarray probe between poor and good responders in both blood and prefrontal cortex. The array probes are ordered according to agglomerative hierarchical clustering, but no large common gene regulation patterns are revealed between the 2 tissues. Scale for color coding difference in expression is identical for (A) and (B). All raw data for Fig 4 are available in S1 Data. FDR, false discovery rate.

Although no robust gene regulation was apparent when the treatment group (independent of response) was compared to the control group, there was a pronounced effect within the treatment group. We were able to detect a set of 259 transcripts that showed a significant difference in expression due to antidepressant response status at a false discovery controlled significance level of 10% (*q* < 0.1) (Fig 4; S1 Table), of which 85 had *q* < 0.05 (S1 Table).

We then aimed to see whether the observed gene regulation patterns in peripheral blood might overlap with effects observed in the PFC from the same animals. To test this, we first performed a cluster analysis on the difference in expression between the responder groups in the set of differentially regulated genes in blood. We then compared the results for these transcripts to the difference in expression between these 2 groups measured in PFC brain tissue in the same animals. The results are summarized in a heat map in Fig 4 and indicate that, within the selected gene set, there is no major common gene regulation pattern associated with response status between both tissues.

### Impact of blood cell proportions in mice gene expression profiles

No significant differences in immune cell subtypes between the different response groups were detected using CIBERSORT [19] and ImmuCC [20] (see S3 Table).

### Impact of blood cell proportions in human gene expression profiles

No significant change in immune cell subtypes using CellCODE [23] was associated with the response groups in the human sample (see S4 Table). Therefore, none of the estimated cell proportions were included in further analyses.

### Murine signature gene expression transcripts predict antidepressant treatment response in the human sample

In the next step, we determined whether this transcriptional profile identified in the mouse model would also be relevant in the human data set. Therefore, we tested whether changes in the mRNA expression of the human orthologues of transcripts at FDR of 10% and at FDR of 5%, separately, are associated with response to antidepressant treatment. Differences in expression profiles from baseline to week 12 when using human orthologues of transcripts at FDR of 10% allowed prediction of response status (at least 50% improvement in HDRS-17 from baseline to week 12 for responders) with an accuracy of 76%, using all patients treated with antidepressant. The prediction persisted after we permuted the response-status labels 10,000 times (*p*_*perm*_ = 0.0328). When a more stringent FDR of 5% cutoff was applied to the mouse transcripts, the corresponding human orthologues predicted response status with an accuracy of 81% in the human sample. The prediction persisted after we permuted the response-status labels 10,000 times (*p*_*perm*_ = 0.0018).

**Fig 5:**
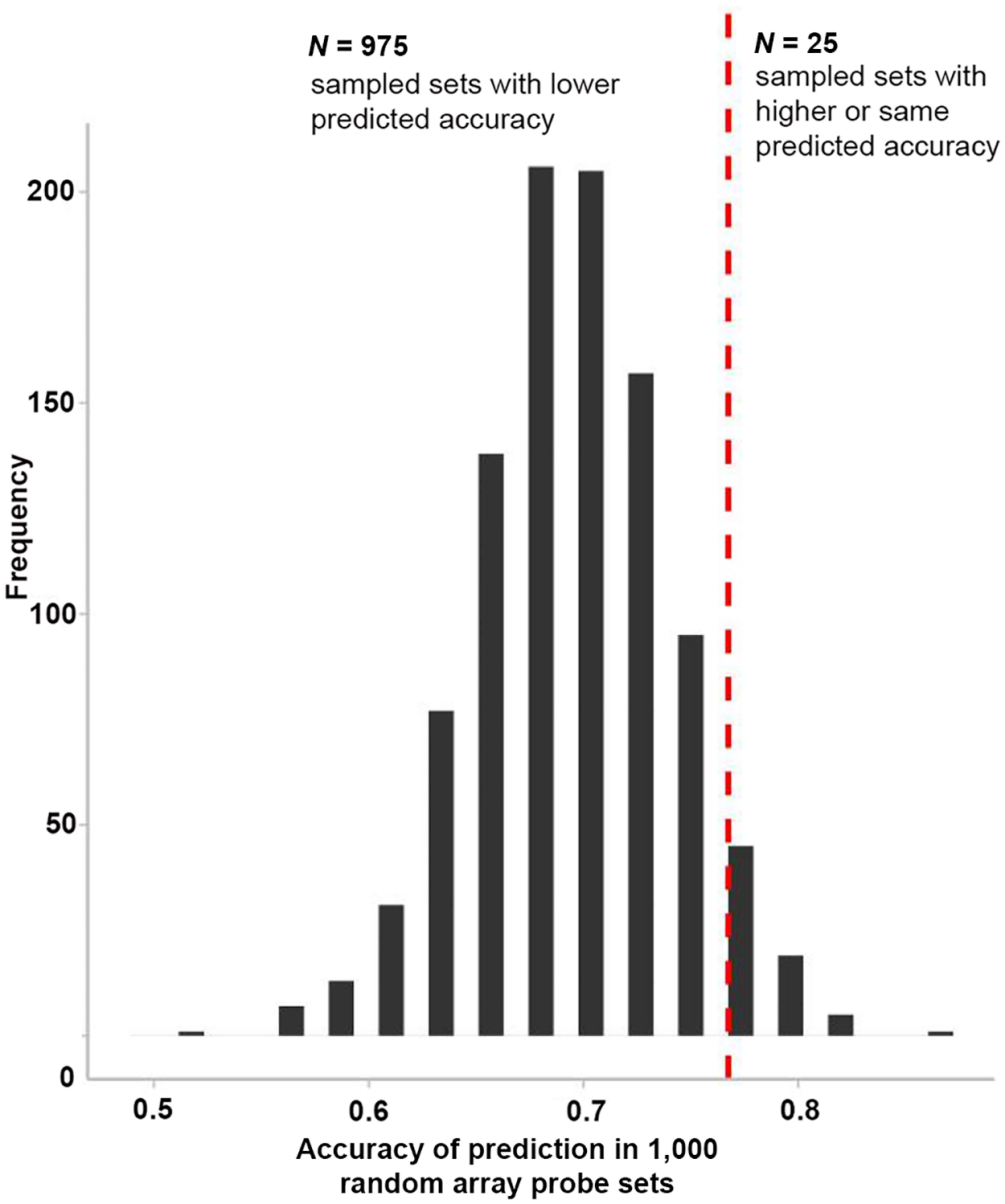
Classification features selected from differential gene expression in a mouse model for antidepressant treatment response are informative for treatment response in a human gene expression data set. Histogram shows the distribution of prediction accuracy over 1,000 simulated classification models that were computed with randomly chosen gene expression probe sets from human gene expression data (median prediction accuracy = 69.77%). The red dashed line marks the observed prediction accuracy (76.74%) when using our informed feature selection to build classifiers. Because only 25 of the randomly chosen feature sets yield equal or better classification results, the predictive ability of our features selected from the presented mouse model is significantly greater than expected by chance (*p* = 0.026). All raw data for Fig 5 are available in S1 Data.

After showing that expression levels of the antidepressant response genes identified from mice are also informative for classification in a human sample, we further analysed the quality of the mouse-based feature selection in the human data set. For this, we compared the classification accuracy of our identified antidepressant-response features to classification accuracy given by randomly chosen and size-matched sets of gene expression probes in the human sample (Fig 5). In analogy to the previous classification approach, we used differences in gene expression from baseline to week 12 in 1,000 random sets of gene probes. Only 25 random gene probe sets showed higher or equal prediction accuracy than our feature panel selected from the animal model. This suggests that the information derived from the mouse experiments allowed the selection of transcripts for which the classification accuracy is better than for random gene expression background (*p*_*perm*_ = 0.026).

### Functional annotation

For functional annotations of the microarray results, we performed pathway analyses and included an overrepresentation analysis with DAVID, and we conducted a second analysis using Pathway-Express. The latter accounts for pathway topology and biological effect size. In both approaches, no significant results passing our threshold criteria were found. The top overrepresented categories in DAVID were entities associated with general gene transcription and did not reach significance levels. Although Pathway-Express showed formally significant results for a few specific Kyoto Encyclopedia of Genes and Genomes (KEGG) pathways, we excluded them because less than 2% of the pathway genes were regulated.

### Genes regulated by dexamethasone are enriched in paroxetine treatment responsive genes

**Fig 6:**
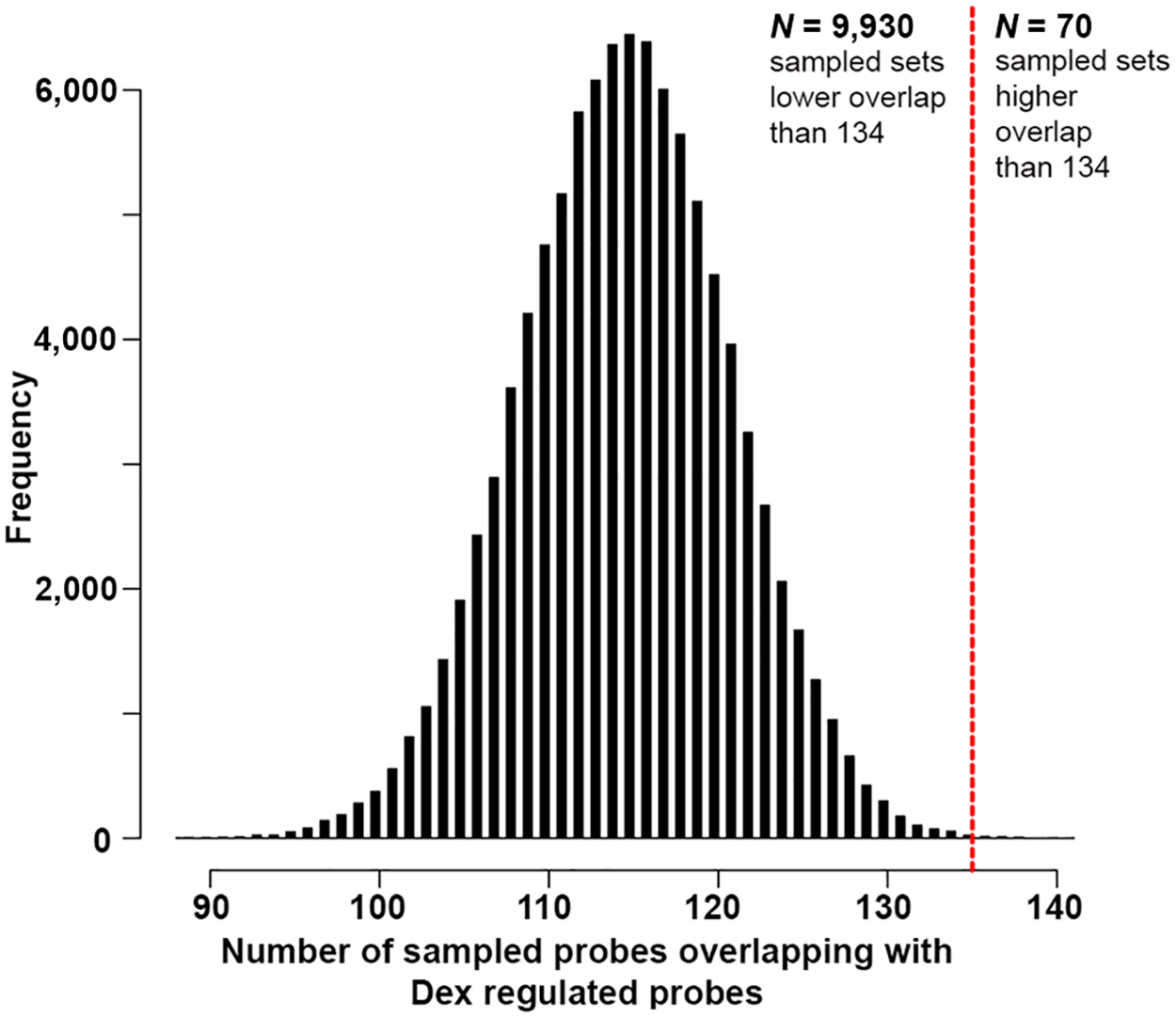
Enrichment of dexamethasone-regulated genes. Histogram of 100,000 random samples indicates that paroxetine response genes show higher overlap with dex-regulated genes than expected by chance. The overlap between differential microarray probes from paroxetine-response and dex-regulated microarrays is 134 and this threshold is indicated as a vertical red dashed line. In this simulation, on average, 115 probes do overlap by chance and in 70 samples, the random overlap is higher than the tested one. All raw data for Fig 6 are available in S1 Data. dex, dexamethasone.

We next integrated our results with another microarray data set that we had previously generated. Those data originated from mouse blood samples taken from animals that had been treated with the glucocorticoid receptor (GR) agonist dexamethasone (dex [25]). To test whether GR activation responsive genes are overrepresented in our antidepressant response gene set, we used a permutation approach and computed the overlap of dex-regulated genes with the paroxetine response genes and compared it to matched random gene sets sampled from the paroxetine array results (Fig 6). Based on 2,852 array probes that constituted a common content for both independent data sets, 179 array probes of the 259 response associated probes could be used for this analysis. The overlap between the probes significantly regulated between the responder group and the ones regulated following dex administration was 134 out of 179. Within 100,000 trials of drawing random gene sets of 179 probes, there were only 70 instances in which a higher overlap occurred. This reflects a permutation-based FDR of 7e-4 for enrichment of dex-regulated array probes in paroxetine response probes. A hypergeometric test yielded a *p*-value of 5.6e-4, further supporting an enrichment of GR-responsive transcripts among response-associated genes.

Standard enrichment analysis does not take into account the direction of gene regulation, and we were interested to see whether paroxetine response and dex regulation showed a directional overlap. Of the 134 array probes that are significantly regulated by dex and are, at the same time, between the paroxetine response groups, only 38 had a mismatch in the direction of the putative regulation. The majority of the regulated genes (*N* = 96) are regulated in the same direction in both conditions, and based on a binomial distribution, such a result could not be observed if both outcomes (same and opposite regulation) had the same probability (*p* = 7.2e-07). Thus, we can conclude that there is a common direction of gene regulation for dex treatment and paroxetine response.

## Discussion

The goal of the present study was to gain insight into the biology of variations in response to antidepressant treatment and to describe molecular signatures associated with response, ultimately aiming at the identification of predictors of treatment outcome. Based on a conceptually novel translational approach, starting with stratification into extreme phenotypes in the mouse, we were able to identify common—i.e., conserved across species—informative transcript sets associated with antidepressant treatment outcome. Intriguingly, we finally show that GR-regulated genes are significantly enriched in this cluster of antidepressant-response genes, pointing to the involvement of GR sensitivity as a potential key mechanism in shaping transcriptional changes and clinical response to antidepressant treatment.

### Modeling heterogeneity of antidepressant response in the mouse: The approach

There are 2 obvious gaps of knowledge in depression treatment, namely (1) the lack of biosignatures predicting antidepressant response and (2) the lack of knowledge of the molecular mechanisms mediating the response to antidepressant pharmacotherapy. The latter is of particular importance for the eagerly awaited discovery of conceptually novel antidepressant treatment strategies, which can only be rationally realized with a deeper understanding of the molecular mechanisms underlying clinical response [26].

In recent years, the unbiased, i.e., genome-wide, screening to identify genetic factors that could assist in the prediction of an individual’s drug response has been a major focus in depression research. Despite tremendous efforts, however, the results are fairly modest in identifying predictive genes in large genome-wide association studies [27–29] and even in a meta-analysis [6]. Instead, Tansey et al. [30] recently presented data implicating a highly polygenic architecture involving many common variants scattered across the genome, none of which have very large effects but cumulatively contribute to a substantial proportion of variation in antidepressant response. So far, only a few small studies provided first evidence that biochemical information (e.g., metabolomics) could add to the panel of markers predicting response to a particular antidepressant in patients [31], suggesting that alternative strategies need to be explored.

However, studies to investigate the neurobiology of antidepressant treatment response have been hampered by the fact that no appropriate animal model addressing this issue had yet been described. Therefore, we embarked upon the development of an animal experimental approach modeling the heterogeneity in response to antidepressant treatment as closely as possible. In contrast to studies in patients, this model approach both enables an in-depth analysis of the neurobiological mechanisms shaping individual antidepressant response in the central nervous system and searches for peripheral biosignatures associated with treatment response. There are different approaches to model depression-like phenotypes (i.e., symptoms of depression) in the mouse. While induction of depression-like symptoms following exposure to different types of stress, e.g., chronic social defeat or chronic mild stress is one possible approach, the use of mouse strains with high innate anxiety- and depression-like behavior is also commonly accepted. The selection of the DBA/2J mouse strain, with its well-described high innate anxiety and responsiveness to antidepressant treatment [17], enabled us to perform the pharmacological treatment under basal conditions, i.e., without the need to subject the animals to an additional stress procedure that might have influenced the transcriptome data. A combination of stress exposure and antidepressant treatment within our approach would not allow us to identify the individual contribution of these 2 factors to the phenotype. Nonetheless, a comparison of stress-related and antidepressant response–related molecular events could enable the identification of shared molecular pathways.

Oral treatment with the SSRI paroxetine significantly reduced—as expected—depression-like behavior. Remarkably, in addition to the overall antidepressant-like effect on promoting active coping strategies in the FTS, we detected a high variability in the behavioral outcome. Although the neurobiological mechanisms underlying antidepressant-induced behavioral changes in the FTS still are not fully understood [32], we here used the FST as the laboratory animal equivalent of treatment response because it is the most commonly used test to screen for antidepressant efficacy in rodents [16]. Comparable approaches for stratification and extreme case sampling in animal models have been successfully introduced in the field of stress research [33], and during recent years, they have enabled the identification of a number of key mechanisms shaping individual susceptibility to stress [34, 35]. We considered plasma paroxetine concentration as a covariate on our microarray analyses, but we were not able identify a significant influence on the gene expression profile associated with treatment response.

The selection of a rodent approach for biomarker discovery in psychiatric disorders has the advantage of minimizing potentially confounding variables, which, in clinical depression studies, so far have impeded biomarker discovery [12]. Due to the standardized experimental conditions, factors such as sex, age, and additional environmental factors, including pharmacological pretreatment, the time of day at which the blood sample is taken, physical exercise, food, and many others [36], can be strictly controlled for, thus enabling the detection of true response biomarkers in a hypothesis-free approach. In a second step, these murine biomarkers can then be validated in the human population. Given the complexity of identifying true biomarker candidates in psychiatric disorders, the need to strengthen potential candidates by cross-species approaches [37] and to validate those in independent cohorts is considered crucial [38].

### Translation to the patient: Murine signature gene expression transcripts predict antidepressant treatment response in the human sample

Aiming to enable a translational approach, we focused on the identification of transcriptome signatures in the periphery, because only those are relevant for clinical application. Several studies have investigated the use of human peripheral blood cells as surrogate material for different organs and tissues, including the central nervous system [39–41]. However, inconsistent results have been reported as to the overlap between transcriptome profiles in peripheral blood and brain [14].

To address issues of cross-tissue relevance, we compared peripheral transcriptome signatures with expression profiling data of the PFC of the same good- and poor-responding animals. We did not find any major common response status-associated gene regulation pattern between both tissues. We thus hypothesize that in depression treatment, blood cells might act as sentinels of treatment response but are not generally informative about central regulation processes, at least not in the PFC.

In the next step and as a proof of concept, we sought to evaluate the relevance of the murine transcriptional signature associated with antidepressant treatment response in a human data set. Using a powerful within-participant approach investigating longitudinal transcription changes between baseline and week 12 of antidepressant treatment, we tested whether mRNA expression of the human orthologues of these transcripts changes with antidepressant treatment in peripheral blood in a subset of 2 human studies [21, 22]. Differences in expression profiles from baseline to week 12 of the human orthologues selected on the basis of the murine transcript signature allowed prediction of response status (percent change in HDRS-17 from baseline to week 12) with an accuracy of 76% in the human sample. Using a permutation strategy, we also showed that our set of transcripts was more likely to predict treatment outcome correctly than random sets of transcripts. We thus show the suitability of an appropriate animal experimental approach for the discovery of peripheral treatment response biomarkers. While promising, our findings certainly require validation in independent samples of patients with MDD. One aspect that needs more detailed investigation in future studies is the precise time course and stability of response-associated transcript changes, as we here integrated murine transcript data following 2 weeks of antidepressant treatment with patient data over a 12-week treatment course.

### Modulation of GR sensitivity as underlying mechanism shaping response to antidepressant treatment

The available evidence makes a compelling case implicating dysregulation of the stress hormone system, the so-called hypothalamus-pituitary-adrenocortical (HPA) system, in the pathogenesis of MDD [42, 43]. Moreover, considerable evidence has accumulated suggesting that normalization of the HPA system might be the final step necessary for stable remission of the disease [44], and it was further hypothesized that antidepressants may act through normalization of the HPA system function [45]. A recent study provided evidence that hormone-independent activation of the GR is involved in the therapeutic action of fluoxetine [46], supporting the neurobiological link between GR signalling and antidepressant action.

We could not detect any difference in corticosterone plasma concentrations between good and poor responders to paroxetine treatment directly after the FTS challenge, although assessment of plasma corticosterone concentrations at 1 time point, i.e., 5 min after the FST, does not exclude potential dynamic changes in HPA system response (i.e., changes in the rise of corticosterone or HPA system feedback following initial activation). Evidence from measurements of HPA system activity in depressed patients, however, supports the notion that in vivo challenges such as the combined dexamethasone/corticotropin releasing hormone challenge test (Dex-CRH test) are superior to single baseline measurements of peripheral glucocorticoid concentrations in discriminating between depressed patients and healthy controls as well as treatment responders versus nonresponders. In addition, recent investigations have shown that dex-stimulated gene expression is a sensitive marker of GR-resistance in MDD [13] and that common genetic variants that modulate the initial transcriptional response to GR activation increase the risk for depression [25]. Therefore, we tested for an enrichment of GR-responsive genes in our antidepressant response gene set, a finding that could point to increased GR sensitivity in good- versus poor-responding animals. We demonstrated that (1) GR-regulated genes are significantly enriched in our cluster of antidepressant-response genes and (2) there is a common direction of gene regulation for dex treatment and paroxetine response. Our data are in line with a large body of previous evidence pointing to the normalization of GR resistance as an important feature of the clinical response to antidepressant treatment [43, 47] and support the intriguing hypothesis that antidepressants could stimulate resilience-promoting molecular mechanisms [48].

### Conclusion and perspectives

Biomarkers or biosignatures, respectively, would not only allow monitoring of antidepressant treatment response in clinical practice but they also could assist in the evaluation of drug actions at an early stage in clinical trials of novel agents that are frequently marred by late attrition [49]. In particular, identifying biomarkers of response will be essential for assessing target engagement of novel mechanisms. We submit that our approach opens up the opportunity to generate a unique database for putative biosignatures predicting response to be assessed and validated in larger patients’ samples.

In conclusion, we expect this translational approach to serve as a template for the discovery of improved and tailored treatment modalities for depression in the future.

## Supporting information

S1 Data Contains raw data for Figs 4, 5 and 6 and human and mouse microarray data used in this manuscript. (XLSX)

S2 Data Contains raw data for Fig 3, S1, S2 and S3 Figs. (XLSX)

S1 Table Shows significantly regulated genes in the peripheral blood between good and poor responders after 14 d of paroxetine treatment. Genes are ordered according their functional classes. Fold changes are normalized to poor responders.
(XLSX)

S2 Table Characteristics of MDD patients in human sample. MDD, major depressive disorder.
(DOCX)

S3 Table Impact of blood cell proportions in mouse gene expression profiles. (DOCX)

S4 Table Impact of blood cell proportions in human gene expression profiles. (DOCX)

S1 Fig Neuroendocrine, physiological, and behavioral parameters following treatment with low-dose paroxetine (1 mg/kg BW, twice daily). (A) Twenty-eight days of 1 mg/kg BW paroxetine treatment led to an increase in body weight in the paroxetine-treated animals. (B) Corticosterone levels were not altered due to the treatment. (C) Paroxetine treatment led to a trend in reducing the time spent floating in the treated animals compared to the control group. (D) Chronic treatment did not alter the time spent struggling in the paroxetine-treated group. * significant correlation, *p* < 0.05. All raw data for S1 Fig are available in S2 Data. BW, body weight.
(TIF)

S2 Fig Impact of 14-d paroxetine treatment on physiological and neuroendocrine parameters. (A) After 14 d of paroxetine treatment, animals treated with the SSRI gained significantly more body weight compared to the vehicle-treated control group. (B) No significant difference in body weight gain was found between the responder groups due to the paroxetine treatment. (C) Corticosterone levels were assessed in blood plasma during the circadian nadir in the morning directly after the FST. We did not find any significant difference in corticosterone levels between vehicle- and paroxetine-treated animals. (D) While comparing the corticosterone levels in the different responder groups, no difference was found between the groups. Data are represented as mean + SEM. * significantly different from vehicle treated animals, p < 0.000. All raw data for S2 Fig are available in S2 Data. FST, forced swim test; SEM, standard error of the mean; SSRI, selective serotonin reuptake inhibitor.
(TIF)

S3 Fig Paroxetine brain and plasma concentrations following 14 d of antidepressant treatment. (A, B) ANOVA analysis showed a significant association of responder status with both plasma and paroxetine concentrations. In the post hoc analyses, only brain tissue concentrations of paroxetine showed a significant difference between good responders and poor responders. (C) Paroxetine brain and plasma concentrations were closely correlated (*r* = 0.94). All raw data for S3 Fig are available in S2 Data.
(TIF)

## Abbreviations

CRH: corticotropin releasing hormone
dex: dexamethasone
FDR: false discovery rate
FST: forced swim test
GR: glucocorticoid receptor
HDRS: Hamilton Depression Rating Scale
HPA: hypothalamus-pituitary-adrenocortical
KEGG: Kyoto Encyclopedia of Genes and Genomes
MDD: major depressive disorder
PFC: prefrontal cortex
SSRI: selective serotonin reuptake inhibitor
