# Supplementary material for: Common genes associated with antidepressant response in mouse and man identify key role of glucocorticoid receptor sensitivity
Source: PLoS Biol. 2017 Dec 28;15(12):e2002690. doi: 10.1371/journal.pbio.2002690 (PMC5746203; doi:10.1371/journal.pbio.2002690)
Supplement: S2 Table — MDD, major depressive disorder. (DOCX) [file pbio.2002690.s004.docx]

**S2 Table: Characteristics of MDD patients in human sample (n=86)**
