# Supplementary material for: Common genes associated with antidepressant response in mouse and man identify key role of glucocorticoid receptor sensitivity
Source: PLoS Biol. 2017 Dec 28;15(12):e2002690. doi: 10.1371/journal.pbio.2002690 (PMC5746203; doi:10.1371/journal.pbio.2002690)
Supplement: S3 Table — (DOCX) [file pbio.2002690.s005.docx]

**S3 Table: Impact of blood cell proportions in mouse gene expression profiles**

| **Supplementary Table 3** | |
| --- | --- |
| **Cell Type** | **p-value** |
| Mast.Cells | 0.24 |
| Neutrophil.Cells | 0.25 |
| Eosinophil.Cells | 0.20 |
| B.Cells.Memory | 0.73 |
| B.Cells.Naive | NaN |
| Plasma.Cells | NaN |
| T.Cells.CD8.Actived | 0.64 |
| T.Cells.CD8.Naive | 0.29 |
| T.Cells.CD8.Memory | 0.23 |
| M0.Macrophage | NaN |
| M1.Macrophage | 0.70 |
| M2.Macrophage | 0.24 |
| Treg.Cells | 0.22 |
| T.Cells.CD4.Memory | 0.29 |
| T.Cells.CD4.Naive | 0.24 |
| T.Cells.CD4.Follicular | 0.21 |
| Th1.Cells | 0.49 |
| Th17.Cells | 0.24 |
| Th2.Cells | 0.21 |
| Monocyte | 0.38 |
| GammaDelta.T.Cells | 0.97 |
| NK.Resting | 0.71 |
| NK.Actived | 0.40 |
| DC.Actived | 0.82 |
| DC.Immature | NaN |
