# Supplementary material for: Common genes associated with antidepressant response in mouse and man identify key role of glucocorticoid receptor sensitivity
Source: PLoS Biol. 2017 Dec 28;15(12):e2002690. doi: 10.1371/journal.pbio.2002690 (PMC5746203; doi:10.1371/journal.pbio.2002690)
Supplement: S4 Table — (DOCX) [file pbio.2002690.s006.docx]

**S4 Table: Impact of blood cell proportions in human gene expression profiles**

| **Supplementary Table 4** | |
| --- | --- |
| **Cell Type** | p-value |
| Neutrophil-Resting | 0.52 |
| CD4Tcell-N0 | 0.47 |
| Monocyte-Day0 | 0.91 |
| Bcell-naïve | 0.68 |
| NKcell-control | 0.96 |
| PlasmaCell-FromPBMC | 0.16 |
| CD4Tcell-Th1-restimulated12hour | 0.63 |
| CD4Tcell-Th2-restimulated48hour | 0.84 |
| CD8Tcell-N0 | 0.62 |
| DendriticCell-Control | 0.44 |
| DendriticCell-LPSstimulated | 0.07 |
| MemoryTcell-RO-activated | 0.90 |
| MemoryTcell-RO-unactivated | 0.55 |
