# Supplementary figures and images for: Common genes associated with antidepressant response in mouse and man identify key role of glucocorticoid receptor sensitivity
Source: PLoS Biol. 2017 Dec 28;15(12):e2002690. doi: 10.1371/journal.pbio.2002690 (PMC5746203; doi:10.1371/journal.pbio.2002690)

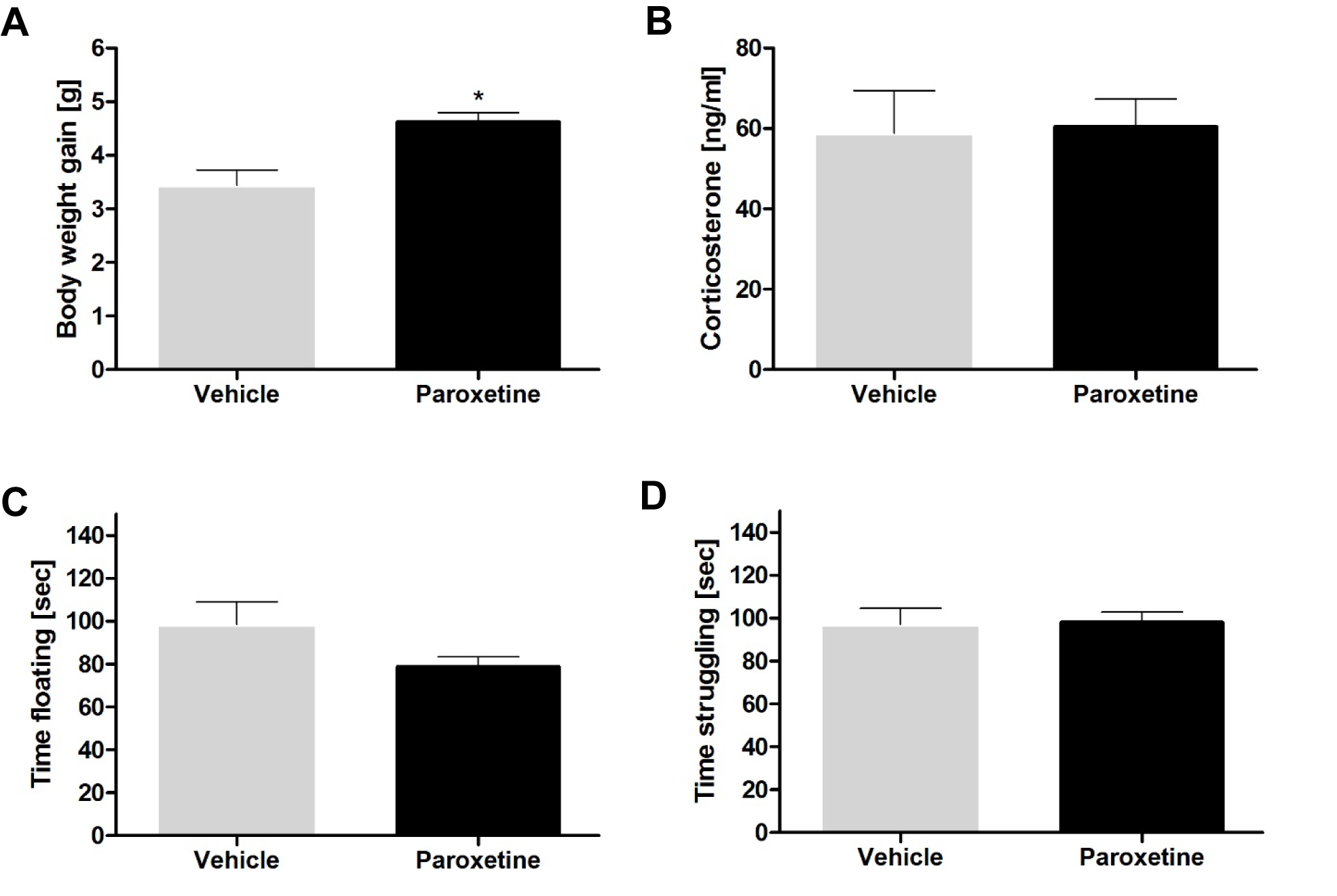

Supplement: S1 Fig — (A) Twenty-eight days of 1 mg/kg BW paroxetine treatment led to an increase in body weight in the paroxetine-treated animals. (B) Corticosterone levels were not altered due to the treatment. (C) Paroxetine treatment led to a trend in reducing the time spent floating in the treated animals compared to the control group. (D) Chronic treatment did not alter the time spent struggling in the paroxetine-treated group. * significant correlation, p < 0.05. All raw data for S1 Fig are available in S2 Data. BW, body weight. (TIF) [file pbio.2002690.s007.tif]

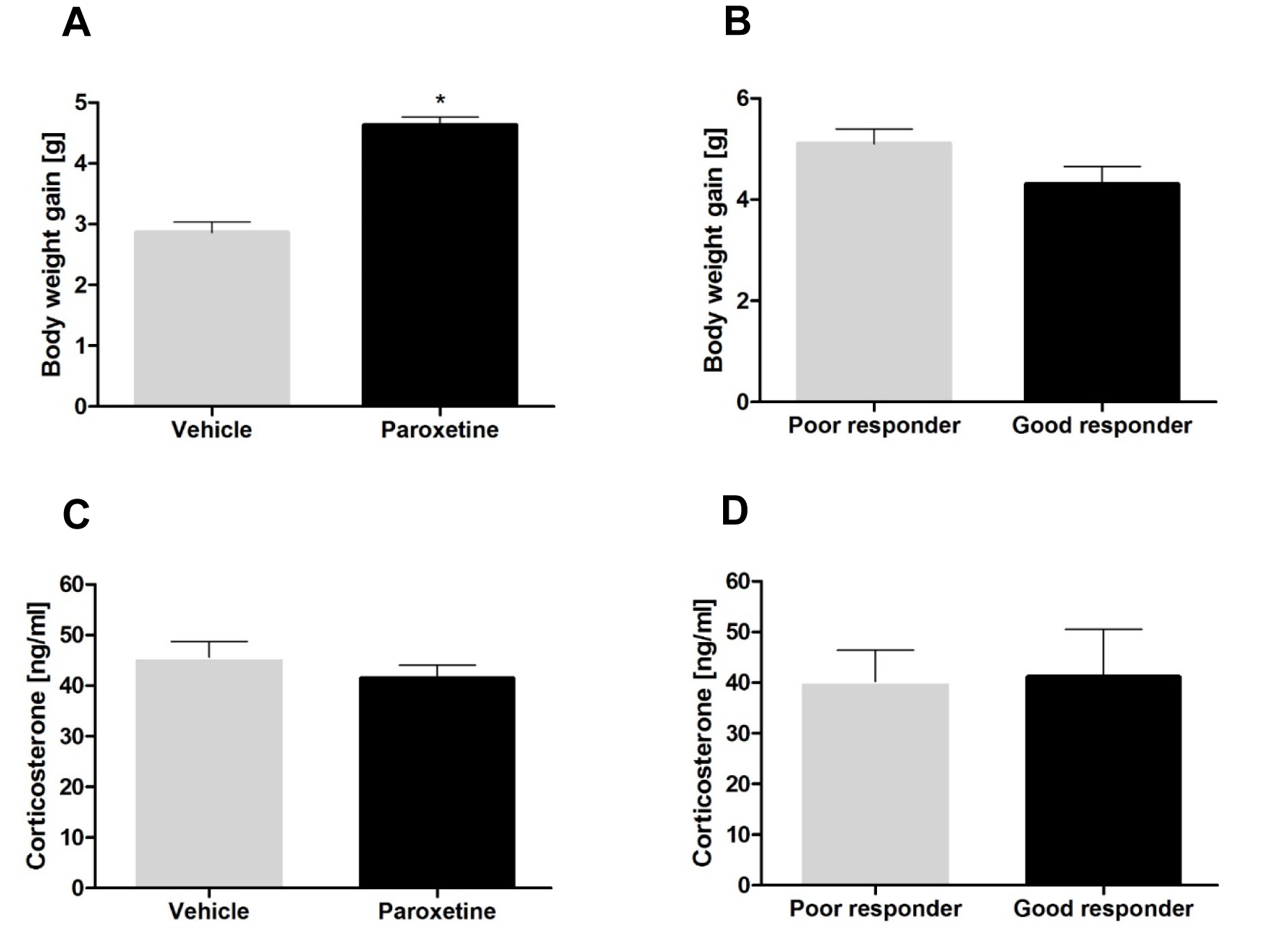

Supplement: S2 Fig — (A) After 14 d of paroxetine treatment, animals treated with the SSRI gained significantly more body weight compared to the vehicle-treated control group. (B) No significant difference in body weight gain was found between the responder groups due to the paroxetine treatment. (C) Corticosterone levels were assessed in blood plasma during the circadian nadir in the morning directly after the FST. We did not find any significant difference in corticosterone levels between vehicle- and paroxetine-treated animals. (D) While comparing the corticosterone levels in the different responder groups, no difference was found between the groups. Data are represented as mean + SEM. * significantly different from vehicle treated animals, p < 0.000. All raw data for S2 Fig are available in S2 Data. FST, forced swim test; SEM, standard error of the mean; SSRI, selective serotonin reuptake inhibitor. (TIF) [file pbio.2002690.s008.tif]

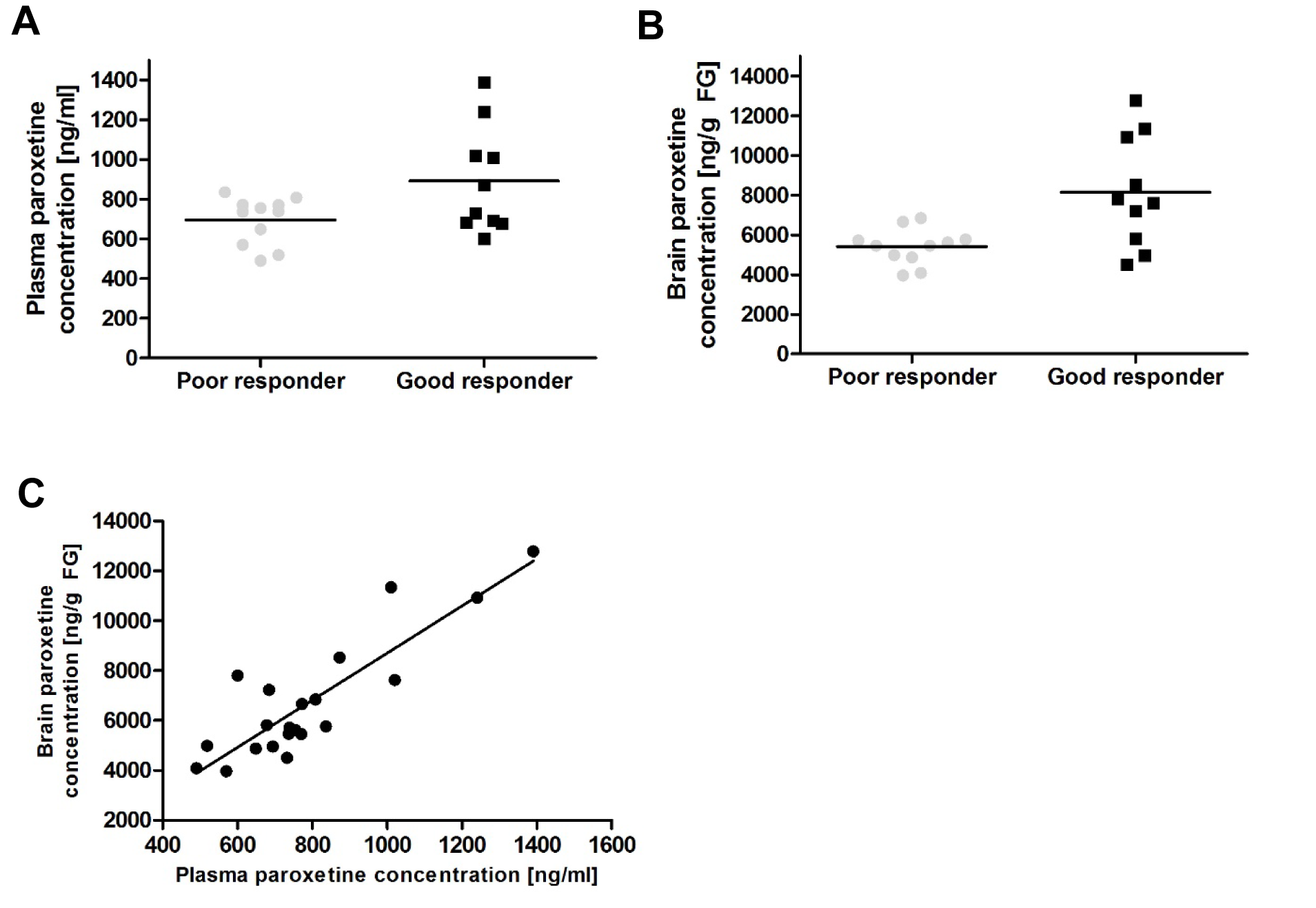

Supplement: S3 Fig — (A, B) ANOVA analysis showed a significant association of responder status with both plasma and paroxetine concentrations. In the post hoc analyses, only brain tissue concentrations of paroxetine showed a significant difference between good responders and poor responders. (C) Paroxetine brain and plasma concentrations were closely correlated (r = 0.94). All raw data for S3 Fig are available in S2 Data. (TIF) [file pbio.2002690.s009.tif]
